# Uterine Lipoleiomyoma: A Case Report and Review of Literature

**DOI:** 10.7759/cureus.20297

**Published:** 2021-12-09

**Authors:** Janat M Alsaif, Zainab S Alali, Tarek Elsharkawy, Ayesha Ahmed

**Affiliations:** 1 Pathology, Imam Abdulrahman Bin Faisal University, Dammam, SAU

**Keywords:** s100, pathology, gynecology, fibroid, leiyomyoma, lipoleiomyomas

## Abstract

Uterine lipoleiomyoma is a rare benign neoplasm that falls under the umbrella of uterine leiomyoma. Upon histological examination, leiomyoma is primarily composed of smooth muscle cells of the uterus. Whereas, lipoleiomyoma reveals a characteristic pattern of mature adipocytes interspersed in uterine smooth muscles. This paper is discussing a case of a 60-year-old postmenopausal grand multiparous woman who presented with postmenopausal bleeding. A primary diagnosis of leiomyoma was made, however, histopathological diagnosis revealed its rare variant, lipoleiomyoma.

## Introduction

Uterine leiomyoma is a benign neoplasm that has been reported to be the most prevalent pelvic mass. The cumulative incidence of leiomyomas in the United States has been estimated to be 70% in Hispanic women and 80% in women of African origin by the age of 50 [1]. The neoplasm’s pathology has been attributed to the hyperproliferation of uterine smooth muscle cells due to the continuous stimulation of circulating estrogen. Thus, its presence and growth peaks during reproductive years and decline after menopause [1]. Uterine lipoleiomyoma is an unusual benign variant of leiomyoma that is characterized by an admixture of fat cells as well as uterine smooth muscle cells on histological examination [2]. The estimated incidence of uterine lipoleiomyoma has been reported to range from 0.03% to 0.2% [3]. Moreover, uterine lipoleiomyomas are more commonly seen in perimenopausal or postmenopausal obese women [2]. The clinical presentation of lipoleiomyoma involves variable signs and symptoms, yet, the vast majority are asymptomatic. When symptomatic, patients may present with a palpable pelvic mass, abdominal pain, abnormal uterine bleeding, and urinary frequency [2]. The diagnosis of lipoleiomyoma primarily relies on a careful history, physical examination, imaging modalities, histopathology, and immunohistochemistry. The treatment options vary; expectant management is usually indicated for asymptomatic women, whereas surgical therapy is usually preferred for large lipoleiomyomas [4]. The early detection of this disease is essential to exclude malignant neoplasms and implement appropriate treatment strategies. This paper presents a case of a 60-year-old Saudi female, diagnosed with lipoleiomyoma.

## Case presentation

A 60-year-old postmenopausal grand multiparous woman presented to the emergency department (ED) with a history of postmenopausal bleeding for a one-week duration. The patient’s last menstrual period was reported to be four years prior to her presentation. A similar episode of postmenopausal bleeding was reported in 2018, in which she sought medical advice; both pap smear and endometrial biopsy were obtained, and results illustrated the absence of malignant cells or atypia. Additionally, her past medical history was significant for osteoporosis secondary to hyperparathyroidism as well as uterine leiomyoma diagnosed 18 years prior to her presentation. Her general assessment and physical examination were unremarkable. However, pelvic examination revealed minimal blood upon the inspection of the vaginal introitus. Moreover, a vaginal swab was obtained and the results were consistent with bacterial vaginosis. A pap smear was also taken and the results were negative for both intraepithelial lesions or malignancy. However, a sufficient endometrial sample could not be obtained. Routine hematologic investigations were all within the normal range. Moreover, pelvic ultrasonography (US) showed a heterogeneous lesion, measuring approximately 5.9 × 3.6 cm, representing subserosal leiomyoma. Imaging also reported an endometrial thickness of 5 mm, and a right ovarian cyst measuring 1.4 × 1.2 cm. Further imaging was not done and a primary diagnosis of subserosal leiomyoma was established. The patient was counseled regarding the available management options, which included total abdominal hysterectomy with bilateral salpingo-oophorectomy (TAHBSO) or hysteroscopy with dilation and curettage (D&C), and the patient preferred to undergo TAHBSO.

**Figure 1:**
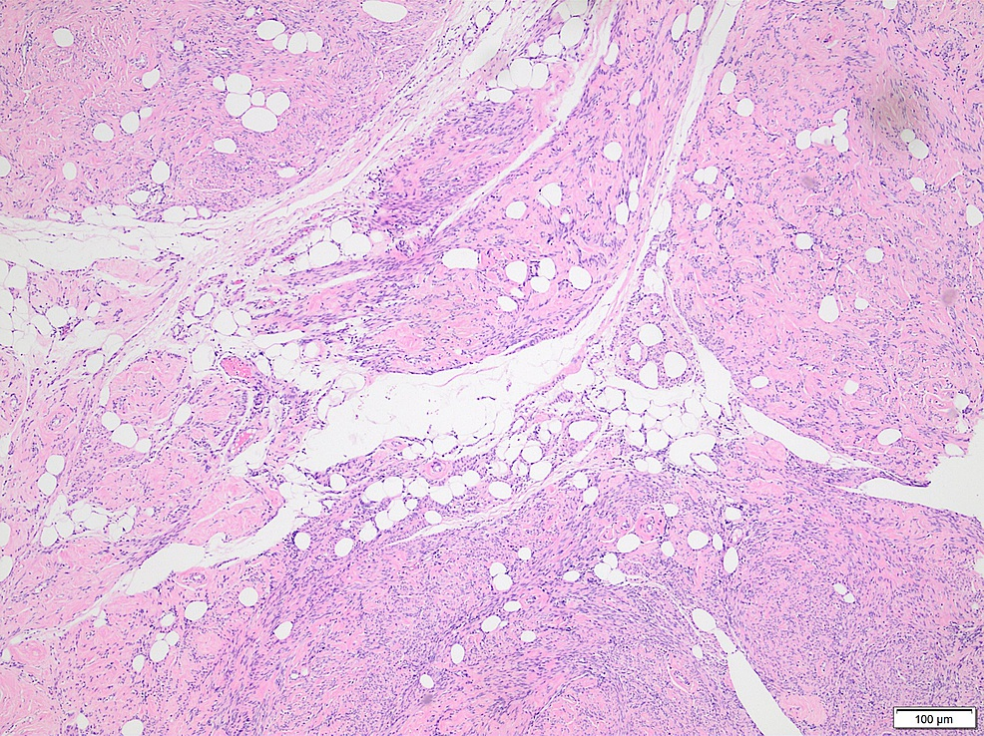
Bundles of smooth muscles with intervening fat cells (H&E, ×200).

**Figure 2:**
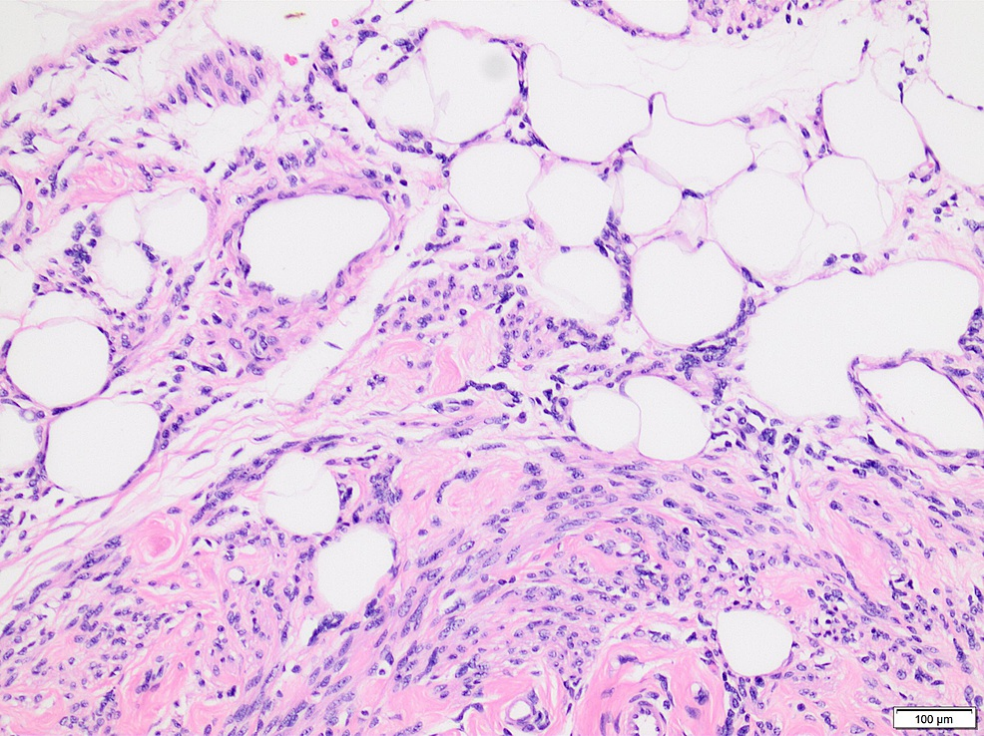
High power of the previous image showing centrally located oval nuclei of the smooth muscle cells and flat nuclei of the fat cells (H&E, ×400).

**Figure 3:**
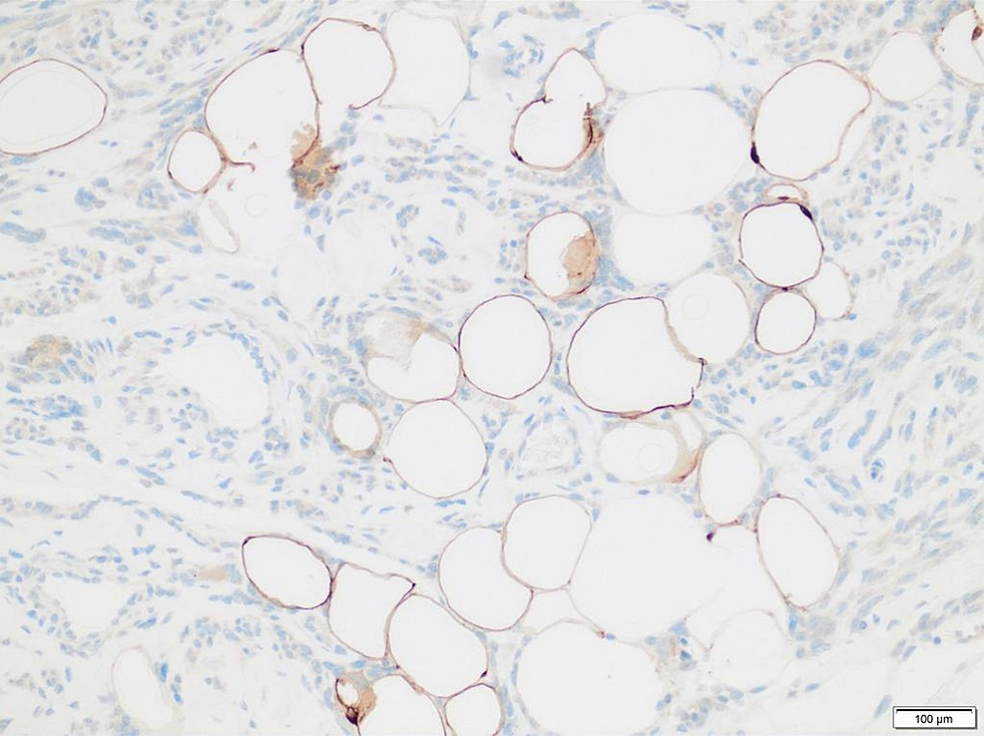
S100 protein highlighting the entrapped fat cells within the leiomyoma (S100 protein stain, ×200).

Post-operatively, the pathology report revealed a gross description of the specimen; two right ovarian cysts were observed; a large whitish cyst and a small translucent cystic lesion measuring 1.5 cm and 0.5 cm in diameter, respectively. According to the pathologists, the endometrial cavity was distorted, measuring 5 cm longitudinally. Nonetheless, the uterine wall was found to have two intramural well-circumscribed fibroids, which were characteristically white tan in color and measuring 5.5 × 3.5 cm and 3 × 3 cm. Cut section of the fibroids showed a whorly pattern, with no identified hemorrhage or necrosis. The left ovary and bilateral fallopian tubes were unremarkable on sectioning. Microscopically, the tumor was composed of bundles of smooth muscle fibers running in various directions and expressing desmin and smooth muscle actin (SMA). In addition to the presence of intervening fat cells that were positive for S100 protein. Moreover, the tumor did not express Human Melanoma Black-45 (HMB-45), which ruled out the rare mesenchymal tumor; perivascular epithelioid cell neoplasm (PEComa). The mentioned microscopic findings were highly suggestive of the rare variant lipoleiomyoma (Figures 1, 2, 3). The patient was discharged from the hospital without complications three days post-TAHBSO with a scheduled outpatient department (OPD) follow-up after four weeks.

## Discussion

Uterine lipoleiomyomas are a very rare sub-entity of uterine leiomyomas, in which the literature reported its incidence to be 0.03% to 0.2% in 1978 [3]. However, the stated results of a recent study conducted in 2014 reported a higher incidence of 2.9% [5]. The mentioned neoplasm is recognized by the presence of abundant and mature adipocytes interspersed in uterine smooth muscle. To our knowledge, its pathogenesis remains uncertain, however, multiple hypotheses have been proposed. This includes fatty infiltration, degeneration, the iatrogenic introduction of adipose tissue to the myometrium, metaplastic transformation of uterine smooth muscle cells into adipose cells, in addition to the transformation of undifferentiated mesenchymal cells [5].

Contrary to the well-known types of leiomyomas, the peak incidence of lipoleiomyomas has been stated to be in perimenopausal or postmenopausal women. A study concluded that 90% of women diagnosed with lipoleiomyomas were above the age of 40 [6]. In addition, the disease was noted to be of higher incidence in obese women [2]. Furthermore, the nature of lipoleiomyomas progression opposes that of leiomyomas, in which it was noticed to continue in its growth even after menopause despite the lack of circulating estrogen [4]. Multiple studies and case reports documented the intramural uterine corpus as the most common location of this benign neoplasm. However, involvement of other sites, such as the cervix and ampulla of the fallopian tube was reported in the literature [7,8].

The diagnosis could be established by clinical presentation, imaging, and histopathology. Clinical symptoms are variant and size-dependent. While most patients are asymptomatic, some may complain of pelvic pain, abnormal uterine bleeding, urinary frequency, and constipation [2,5]. Radiological imaging is of paramount importance for the detection of this tumor, in which the pelvic US may reveal a well-demarcated, hyperechoic lesion surrounded by a hypoechogenic rim [5]. A computed tomography (CT) scan will present low attenuation of the fat component, however, it holds the disadvantage of poor representation of uterine anatomy [5,9]. Magnetic resonance imaging (MRI) is the most beneficial imaging modality, due to its fair representation of detailed uterine anatomy. In the case of lipoleiomyomas, hyperintensities on the T1 weighted sequence are found [5,9,10]. In regards to the histopathological picture, it would represent itself as adipocytes embedded in smooth muscle cells [2]. A study of 72 cases was conducted to assess the immunohistochemical findings seen in lipoleiomyomas. Specimens were stained for vimentin, desmin, S100 protein, estrogen receptor (ER), progesterone receptor (PR), SMA, HMB-45, and Ki-67. Results illustrated that the fatty tissue and smooth muscle components only expressed vimentin, desmin, S100 protein, ER, PR, and Ki-67 [2].

Lipoleiomyoma is a condition of favorable prognosis, and can rarely progress to leiomyosarcoma. Furthermore, treatment protocols are consistent with those of uterine leiomyomas, and treatment of asymptomatic patients is not recommended [5].

## Conclusions

To our knowledge, the literature remains scarce in discussing the pathogenesis, prevalence, prognosis, specific management, and other aspects of uterine lipoleiomyoma. Thus, we recommend conducting high-quality advanced studies that include a fair number of participants. This recommendation would aid in filling the shortfalls related to this topic, and therefore, improve the patient’s outcome, quality of life, and survival rate.
